# A Review of Immunotherapy in Non-Small-Cell Lung Cancer

**DOI:** 10.3390/curroncol31060258

**Published:** 2024-06-17

**Authors:** Mariana Pilon Capella, Steph A. Pang, Marcos A. Magalhaes, Khashayar Esfahani

**Affiliations:** 1 Department of Oncology, Jewish General Hospital, McGill University, Montreal, QC H3T 1E2, Canada; mpilonc@gmail.com (M.P.C.);; 2 Department of Oncology, Hospital Beneficencia Portuguesa de Sao Paulo, São Paulo 01451-010, Brazil; marcos.magalhes@bp.org.br; 3 Department of Oncology, St. Mary’s Hospital, McGill University, Montreal, QC H3T 1M5, Canada

**Keywords:** immunotherapy, non-small-cell lung cancer, squamous cell lung cancer, PD-1, CTLA-4

## Abstract

Cancer immunotherapy in the form of immune checkpoint inhibitors has led to a dramatic increase in the survival of patients with lung cancer across all stages. Over the past decade, the field has experienced rapid maturation; however, several challenges continue to complicate patient management. This review aims to highlight the data that led to this dramatic shift in practice as well as to focus on key challenges. These include determining the optimal therapy duration, managing frail patients or those with brain metastases, addressing the challenges posed by immune-related adverse events, and defining the various patterns of clinical and radiological responses to immunotherapy.

## 1. Introduction 

Lung cancer is the leading cause of death from cancer for both men and women worldwide. In 2022, 253,537 cases were diagnosed in Northern America, 30,000 of which were in Canada [1,2,3]. About 85% of all lung cancers are non-small-cell lung cancer (NSCLC), divided into adenocarcinoma (40–50%) and squamous cell carcinoma (20–30%) subtypes [2]. Of these, 60% are diagnosed at a locally advanced stage and 40% with de novo metastatic disease [2]. In the absence of a driver mutation, the multimodality approach to the treatment of NSCLC relies on chemotherapy, immunotherapy, surgery, and radiation therapy. Notably, immunotherapy has been the cornerstone of the most significant advancements in lung cancer survival over recent decades. This review will provide an overview of the data supporting its use across all NSCLC stages, highlighting key challenges and unresolved questions. As we review the relevant clinical trials, their summary can be found in Table 1. 

## 2. Immunotherapy for Metastatic NSCLC

### 2.1. Single-Agent Immunotherapy

The first trials for anti-program death ligand 1 (anti-PD(L)1) monotherapy were used in the second-line or third-line setting, with encouraging results demonstrating response rates of around 20%, compared to 9–13% for chemotherapy [4,5,6,31]. Following the landmark phase III trial Checkmate-017 comparing nivolumab to docetaxel, the Food and Drug Administration (FDA) approved nivolumab as the first anti-PD-1 agent for NSCLC with PDL-1 ≥ 1% in 2016. Similar results were seen in Checkmate-057 (nivolumab in non-squamous NSCLC) and Keynote-010 (pembrolizumab in advanced NSCLC) [5,32], particularly in PDL-1 > 50% patients who derived a median overall survival (OS) of 14.9 months on pembrolizumab vs. 8.2 months on docetaxel (HR 0.54, 95% CI [0.38–0.77]). Clinical activity for an anti-PD-L1 targeted drug was seen also in the OAK trial, where atezolizumab was compared to docetaxel in squamous and non-squamous histologies and PDL-1 low/undetectable and PDL-1 high subgroups [6].

The benefit of ICI monotherapy is also seen in pivotal first-line phase III studies. These include Keynote-024 and Keynote-042, which demonstrated the superiority of first-line pembrolizumab monotherapy vs. platinum-based chemotherapy in PDL-1 ≥ 50 and PDL-1 ≥ 1 NSCLC patients, respectively. In Keynote-024, PFS benefit was seen for pembrolizumab vs. chemotherapy (7.7 months vs. 5.5 months, HR 0.5, 95% CI [0.39–0.65]), and in Keynote 042, OS benefit was also seen (16.7 months vs. 12.1 months, HR 0.81, 95% CI [0.71–0.93]). The benefit was seen with atezolizumab in the phase III IMpower110 trial for PD-L1 ≥ 1% NSCLC, although in an updated analysis, the OS benefit was no longer statistically significant [8,33]. Other first-line trials comparing anti-PDL1 monotherapy to chemotherapy were negative, likely due to the inclusion of PDL-1 1–50% patients. This included Checkmate-026 (nivolumab in PDL-1 ≥ 5%) [9], MYSTIC (durvalumab monotherapy vs. durvalumab/tremelimumab vs. chemotherapy in PDL-1 ≥ 25%) [10], and JAVELIN Lung 200 (avelumab in PDL-1 ≥ 1%) [11].

No head-to-head phase III trials compare single-agent immunotherapy to immunotherapy–chemotherapy in PDL-1-high patients. A recent cohort study analyzed 3086 advanced NSCLC patients receiving first-line therapy. Among them, 32% (978) received chemoimmunotherapy and 68% (2108) received immunotherapy alone. The study found no significant difference in OS between the groups, but chemoimmunotherapy offered an early survival advantage in both the overall cohort and the high PD-L1 subgroup (≥90%) [34].

Further evidence comes from a network meta-analysis comparing chemoimmunotherapy and different immunotherapy combinations across studies. This analysis included 12 eligible trials with a total of 7845 patients. In patients with high PD-L1 expression, chemoimmunotherapy improved objective response rate (ORR) and PFS compared to single-agent immunotherapy (not dual-agent immunotherapy). However, there was no difference in overall survival across treatment groups regardless of PD-L1 status [35].

### 2.2. Dual Immunotherapy without Chemotherapy

Another FDA-approved first-line option for metastatic NSCLC with PDL-1 ≥ 1% without EGFR/ALK alterations is nivolumab plus ipilimumab. OS benefit was positive in the phase III trial Checkmate 227, which comprised 1739 patients across all PD-L1 expression subgroups and compared ipilimumab/nivolumab vs. chemotherapy. Patients on ipilimumab/nivolumab had better long-term survival with a 4-year OS rate of 29%, compared to 18% for patients on chemotherapy [12].

Tumor mutational burden’s (TMB) validity as a biomarker to select patients for dual ICI remains unclear. In an exploratory analysis of the MYSTIC trial, comparing durvalumab, durvalumab/tremelimumab, and chemotherapy in NSCLC, a TMB ≥ 20 mutations/Mb was significantly correlated with improved OS for durvalumab/tremelimumab vs. chemotherapy (21.9 months vs. 10.0 months, HR 0.49, 95% CI [0.32–0.74]) and was not correlated with PDL-1 expression, suggesting its potential as a predictive biomarker for responsiveness to dual ICI [10]. In Checkmate-227, progression-free survival (PFS) benefit was seen for TMB ≥ 10 mut/Mb vs. chemotherapy (7.2 months vs. 5.5 months, HR 0.58, 97.5% CI [0.41–0.81]), but no significant OS benefit between high-TMB patients and low-TMB patients [12]. The phase III trial NEPTUNE explored TMB by comparing durvalumab/tremelimumab vs. platinum-based chemotherapy in metastatic NSCLC with TMB ≥ 20 mutations/Mb. However, NEPTUNE failed to meet its primary endpoint for OS and the full results have not yet been published [13].

### 2.3. Single-Agent Immunotherapy Combined with Chemotherapy

In PDL-1 1–49% or <1% patient groups, ICI confers a survival benefit when combined with chemotherapy. Chemotherapy likely enhances immune responses by helping in the priming of the immune system [36]. In the phase III KEYNOTE-189 trial, patients with non-squamous NSCLC and a PDL-1 ≥ 50% and PDL-1 1–49% had a statistically significant PFS benefit (HR 0.35, 95% CI [0.25–0.49], and 0.53, 95% CI [0.38–0.74], respectively), while patients with PDL-1 < 1% had a less impressive benefit (HR 0.67, 95% CI [0.49–0.93]) [14]. Even so, based on KEYNOTE-189, carboplatin/pemetrexed/pembrolizumab obtained approval from the FDA in 2018 as first-line therapy for advanced NSCLC without EGFR/ALK alterations [37].

Similar results were seen with anti-PDL-1 drugs combined with chemotherapy in non-squamous NSCLC patients. This was demonstrated in IMpower130 (atezolizumab-carboplatin-nab-paclitaxel vs. carboplatin-nab-paclitaxel), IMpower132 (atezolizumab-pemetrexed-platinum vs. pemetrexed-platinum) and IMpower150 (carboplatin-paclitaxel vs. atezolizumab-bevacizumab-carboplatin-paclitaxel vs. bevacizumab-carboplatin-paclitaxel). Notably, IMpower150 included patients with EGFR/ALK mutations, accounting for 14% of the study cohort. This subgroup was previously assumed to derive minimal benefit from ICI [38].

An exploratory analysis from IMpower150, with a median follow-up of 39.3 months, hinted at an OS advantage when atezolizumab was added to the bevacizumab-carboplatin-paclitaxel regimen. This potential benefit (HR 0.60, 95% CI [0.31–1.14]), suggests that bevacizumab’s counteraction of VEGF-mediated immunosuppression may enhance T-cell responsiveness [18].

Building on these insights, the phase 3 ATTLAS trial further evaluated the efficacy of atezolizumab in combination with bevacizumab and chemotherapy (ABCP) against the standard pemetrexed plus carboplatin or cisplatin in EGFR or ALK-mutated NSCLC who experienced progression following prior TKI therapy. The study’s findings revealed an ORR improvement in the ABCP arm (69.5% vs. 41.9%) and a median PFS benefit (HR 0.62, 95% CI [0.45–0.86]), with the overall benefit increasing as PD-L1 expression rose. However, the OS was similar (HR 1.01, 95% CI [0.69–1.46]) [19].

For advanced squamous NSCLC, the phase III trial KEYNOTE-407 examined first-line pembrolizumab combined with platinum-doublet chemotherapy versus platinum-doublet chemotherapy. The trial met its primary endpoints for PFS and OS. Statistically significant OS and PFS benefits were present in subgroup analyses for PDL-1 1–49%. There was also a positive trend in the PDL-1 < 1 subgroup [20]. As a result of KEYNOTE 407, pembrolizumab with chemotherapy was approved in 2018 by the FDA for first-line treatment of metastatic squamous NSCLC [33]. Meanwhile, atezolizumab is not approved for the squamous population; the phase III IMpower131 trial (atezolizumab plus chemotherapy vs. chemotherapy in metastatic squamous NSCLC) demonstrated statistically significant benefit for PFS, but not for OS [16].

### 2.4. Dual Immunotherapy Combined with Chemotherapy

The phase III Checkmate-9LA trial evaluated ipilimumab/nivolumab with two cycles of chemotherapy. OS benefit was seen in PDL-1 ≥ 1% and PDL-1 < 1% in patients receiving ipilimumab/nivolumab with chemotherapy compared to chemotherapy alone (15.8 months vs. 10.9 months, HR 0.74, 95% CI [0.6–0.93], and 17 months vs. 9.8 months, HR 0.67, 95% CI [0.51–0.88], respectively) [21]. In the phase III POSEIDON trial for mNSCLC, the combination of tremelimumab plus durvalumab and chemotherapy (T + D + CT) and durvalumab plus chemotherapy (D + CT) were evaluated against chemotherapy alone (CT). The study found that D + CT improved PFS compared to CT alone (HR 0.74, 95% CI [0.62–0.89]). Although the OS improvement trend for D + CT was not statistically significant (HR 0.86, 95% CI [13.3 vs. 11.7]), adding tremelimumab to the mix (T + D + CT) improved both PFS (HR 0.72, 95% CI [0.60–0.86]) and OS (HR 0.77, 95% CI [0.65–0.92]) when compared to CT. Regarding PD-L1 levels, in the POSEIDON trial, a statistically significant OS and PFS benefit was present in subgroup analyses for PDL-1 50% for both D + CT and T + D + CT, with a positive trend for OS in the PD-L1 > 1% subgroup in both arms [22].

## 3. Optimal Duration of ICI Therapy in Advanced Disease

Trials with ipilimumab and nivolumab (e.g., CheckMate-9LA), the KEYNOTE trials with pembrolizumab (e.g., 024, 042, 189, and 406) and the EMPOWER trials with cemiplimab (e.g, Lung 1 or 3) set a limit of two years, 35 cycles, and 108 weeks, respectively. There was no limit in the IMpower trials (e.g., 130 or 150) with atezolizumab or in the POSEIDON trial with durvalumab (although a maximum of 5 doses of tremelimumab was administered in combination with durvalumab). Checkmate 153, a phase III/IV real-world population study that evaluated the safety and efficacy of nivolumab in previously treated patients with stage IIIB or stage IV NSCLC, sought to answer the question of the optimal duration of ICI therapy [23]. The protocol was amended to randomly assign patients to either 1 year of nivolumab therapy or until progression. A treatment with duration beyond one year conferred a significant survival advantage compared to an early stoppage (PFS 24.7 months vs. 9.4 months; hazard ratio [HR], 0.56 [95% CI, 0.37–0.84]). Despite limitations such as small sample size, the unplanned nature of the analysis, and insufficient power to demonstrate a statistically significant survival advantage, this research offers the only prospective data evaluating the relationship between therapy duration and survival outcomes. Further analysis, presented in a retrospective cohort study utilizing the Flatiron Health EHR database and involving 113 patients on fixed duration and 593 on indefinite ICI therapy, revealed no significant difference in survival between the two groups (univariable HR 1.26, 95% CI [0.77–2.08]; multivariable HR 1.33, 95% CI [0.78–2.25]). These findings suggest that extending ICI treatment beyond two years may not provide a significant OS benefit, offering reassurance for those considering discontinuing therapy at the two-year threshold.

Although lacking randomized clinical trials, these analyses offers an unprecedented insight into an unanswered yet clinically significant issue. Some promising biomarkers such as minimal residual disease and measuring circulating tumor DNA are under study to guide the optimal duration of therapy [39,40,41].

## 4. Immunotherapy for Earlier-Stage Resectable NSCLC

Phase II trials of neoadjuvant immunotherapy demonstrated benefits for OS, PFS and pathologic complete response (pCR) of 10–33%, with NADIM (neoadjuvant nivolumab and chemotherapy) [42], NEOSTAR (nivolumab/ipilimumab vs. nivolumab) [43] and LCMC3 (neoadjuvant and adjuvant atezolizumab) [44]. Most recently, Checkmate-816, a phase III trial of patients with stage IB to IIIA resectable NSCLC, randomized patients to receive neoadjuvant nivolumab plus platinum-based chemotherapy or platinum-based chemotherapy alone, followed by resection. The addition of nivolumab resulted in increased pCR rates (24% vs. 2%, odds ratio 13.94; CI [3.49–55.75]) and PFS (HR 0.63, CI [3.49–55.75]) [25]. Based on Checkmate-816, the FDA approved Nivolumab in 2022 as the first neoadjuvant checkpoint inhibitor for resectable NSCLC that is ≥4 cm or is associated with positive lymph nodes [45].

In the perioperative setting, there are data from the AEGEAN [28] (durvalumab and chemotherapy), Neotorch [30] (toripalimab and chemotherapy), Keynote-671 [46] (pembrolizumab and chemotherapy), and Checkmate 77T [27] trial (nivolumab and chemotherapy). In the Keynote-671 trial, neoadjuvant pembrolizumab combined with cisplatin-based chemotherapy significantly enhanced event-free survival compared to chemotherapy alone (HR 0.58 95% CI [0.46 to 0.72]). This indicates a 42% reduction in the risk of these events for the pembrolizumab group. Major pathological response rates were notably higher in the pembrolizumab group (30.2% vs. 11.0%; 95% CI, 13.9 to 24.7). A recent OS update demonstrates that median OS was not reached (95% CI NR-NR) in the pembrolizumab group compared to 52.4 months (95% CI 45.7-NR) in the placebo group, with a HR 0.72 (95% CI, 0.56–0.93; *p* = 0.00517) [29].

The AEGEAN study presented the durvalumab combination as significantly extending event-free survival, with a HR of 0.68 (95% CI, 0.53 to 0.88, *p* = 0.004), translating to a 32% reduction in risk. The pathological complete response was achieved more frequently with durvalumab than with placebo (17.2% vs. 4.3%; 95% CI, 8.7 to 17.6; *p* < 0.001). The OS data are not yet published.

Checkmate 77T’s interim analysis highlighted neoadjuvant nivolumab plus chemotherapy’s benefit, improving event-free survival with a HR of 0.58 (97.36% CI, 0.42–0.81), suggesting a 42% reduction in the risk of progression or death. The pCR rates were significantly higher in the nivolumab group (25.3% vs. 4.7%; odds ratio, 6.64; 95% CI, 3.40–12.97), demonstrating enhanced tumor response pre-surgery. The OS data are also awaited.

In the Neotorch trial, perioperative toripalimab markedly improved EFS with a HR of 0.40 (95% CI, 0.277–0.565; *p* < 0.001), indicating a 60% reduction in the risk of disease progression or death. The MPR and pCR rates were significantly elevated in the toripalimab arm (48.5% vs. 8.4% and 24.8% vs. 1.0%, respectively), underscoring the potent anti-tumor activity of the regimen. The OS data are not yet released.

In the adjuvant setting atezolizumab has demonstrated activity in the phase III IMpower010 trial and also with pembrolizumab in the phase III Keynote-091 trial [26]. In the IMpower010 study, 16 cycles of adjuvant atezolizumab was associated with improved disease-free survival (DFS) in the PDL-1 ≥ 1% subgroups (HR 0.66, 95% CI [0.50–0.88]), but not in the PDL-1 < 1% subgroup [47]. Similarly, in the Keynote-091 study, pathologically confirmed stage IB tumors (≥4 cm) received 18 cycles of adjuvant pembrolizumab with benefit in DFS (HR 0.76 95% CI [0.63–0.91]) [26].

## 5. The Effect of Radiation Therapy on Immunotherapy

Radiation therapy may have an impact on priming the immune system and enhancing immune response, as does chemotherapy. In the KEYNOTE-001 phase I trial, compared with the patients who received pembrolizumab only, the patients who received radiotherapy as a precondition prior to pembrolizumab showed a significantly longer PFS (4.4 vs. 2.1 months) and OS (10.7 vs. 5.3 months) [24]. Similarly, in the randomized phase II PEMBRO-RT trial, patients who received stereotactic body radiation therapy (SBRT) to a single metastatic site within 7 days before initiation of immunotherapy showed an increase in the overall response rate (18% vs. 36%) and disease control rate (40% vs. 64%) at 12 weeks, as well as improved median PFS (1.9 vs. 6.6 months) and median OS (7.6 vs. 15.9 months), compared to those who did not [48].

The effect witnessed in PEMBRO-RT might be in part due to the abscopal effect. This phenomenon is characterized by tumor regression of untreated metastatic disease outside the target of local therapy [49,50]. This effect is defined as a size reduction of 30% in a non-irradiated metastasis, irrespective of other lesions [51,52]. This effect remains rare, and its incidence is not well understood. As one example, a study in Germany screened 168 patients with metastatic disease, including NSCLC patients, to evaluate the abscopal effect during simultaneous irradiation and anti-PD-1 therapy. The effect was observed in 29% of all patients. Three of the seven patients with NSCLC had an abscopal effect [53].

Other studies have shown improvement in patient outcomes with the addition of radiation therapy either before the initiation of immunotherapy or after its failure. These studies are small, non-randomized, and serve the role of hypothesis generation. Currently, numerous phase 1–3 trials of ICI with radiation therapy are underway, as reviewed elsewhere [54].

## 6. Special Populations: Frailty, Brain Metastases and Hyper-Progressors

### 6.1. Immunotherapy for Frail Patients with NSCLC

Aging is thought to be associated with decreased responsiveness to new antigens, low-grade inflammation, defective T-cell memory responses, decreased naïve T cells, and increased susceptibility to autoimmunity [55].

A pooled analysis of KEYNOTE010/024/042 (pembrolizumab monotherapy) demonstrated that patients over 75 years old still had an OS benefit, particularly if PDL-1 ≥ 50% [7]. This was also noted in another pooled analysis of phase III monotherapy trials spanning nivolumab, pembrolizumab, and atezolizumab [56]. However, in subgroup analyses for trials combining pembrolizumab with chemotherapy, patients older than age 65 had less benefit, particularly with squamous NSCLC [57]. In another pooled analysis of phase III data, patients 65 and older with advanced NSCLC, including those ≥75 years, seem to derive similar survival benefits from treatment with PD (L)-1 therapies as patients <65 years of age. Patients 75 and older enrolled in these trials appear to tolerate ICI and have a similar incidence of grade 3 or 4 irAEs compared to the subgroup of patients <65 years of age [56]. Although the incidence of irAEs was similar, the duration of steroid treatment is often longer in this patient population, which can lead to worse geriatric outcomes including delirium and fractures [58]. A limitation of these data is that older patients enrolled in clinical trials are often fitter than what is seen in real practice.

### 6.2. Patients with Brain Metastases

It is estimated that about one-third of newly NSCLC diagnosed patients may develop central nervous system (CNS) metastases throughout the disease, and 20% present with brain metastasis at the time of diagnosis [59]. Patients with CNS disease have mostly been excluded from large registration trials. However, data from smaller phase 2 studies as well as real-world evidence suggest that ICI monotherapy can induce objective intracranial responses on the order of approximately 30%, with higher responses seen in PDL positive tumors [60,61,62,63]. As an example, in a phase II trial of patients with NSCLC with untreated brain metastases 11 of 37 (30%) patients with PD-L1-positive tumors experienced objective CNS responses with pembrolizumab whereas that number was 0 out of 5 (0%) in those with PD-L1-negative tumors [62]. Responses were mostly concordant with extracranial objective responses in nearly 80% of cases that were evaluable for both CNS and systemic responses. The concordance of responses was also seen in a retrospective study of 255 patients treated in an ICU, in which only 13% discordant cranial–extracranial responses were seen. Analysis of the nivolumab expanded-access program in Italy identified 372 squamous NSCLC patients, of whom 38 had asymptomatic brain metastases [63]. The disease control rate in this population was 39%. Median PFS and OS in brain metastasis patients were 5.5 and 6.5 months, respectively. A preliminary presentation of pooled data of patients enrolled in one of five treatment studies with atezolizumab suggests that this drug also has some CNS activity [64]. Furthermore, in one of the trials included in this analysis, among patients with baseline brain metastases, there was a nonsignificant trend toward reduction in the risk of developing new CNS lesions with atezolizumab compared with docetaxel (hazard ratio [HR] 0.42, CI [0.15–1.18]). Although initial data are promising, we await further studies to determine the optimal sequencing and combination therapies in patients with NSCLC and CNS metastasis.

### 6.3. Disease Response Assessment/Hyperprogressive Disease

Patients on ICI can experience different tumor responses than what was seen on chemotherapy. The guidelines of the Response Evaluation Criteria in Solid Tumours (RECIST) working group were updated for response assessment on immunotherapy (iRECIST) [65]. Pseudoprogression, delayed responses and hyperprogressive disease are new types of responses incorporated into the iRECIST guidelines [65].

Pseudoprogression is extremely rare. About 5% of patients on ICI or less will experience a transient increase in tumor volume, resulting from an influx of inflammatory cells into tumor sites, which is then followed by a real response and decrease in tumor burden [66,67,68,69]. Clinical outcomes in patients with pseugoprogression are better, with longer OS compared to patients with progressive disease (median OS not reached vs. 6.4 months; *p* = 0.001) [70,71].

Meanwhile, the definition of hyperprogressive disease remains controversial [72]. Several definitions have been proposed, such as a tumor growth rate being at least twofold greater during ICI than immediately before the start of therapy, or a disease progression of >50% at the time of the first evaluation as compared to the onset of the treatment [73,74]. Essentially, it is characterized by a fast and unexpected accelerated tumor progression, suspected to be induced by ICI [75]. Hyperprogressive disease has been reported in 6–29% of advanced cancer patients [76]. In NSCLC, the exact incidence is unknown but is thought to be low. Studies showed that patients who were treated with immunotherapy and developed hyperprogressive disease had a worse prognosis, with significantly inferior PFS and OS compared to patients on chemotherapy. As an example, a retrospective study analyzed 335 patients with advanced NSCLC treated with PD(L)-1 monotherapy [77]. A total of 135 had PD by RECIST 1.1 criteria, 44 of which were found to have hyperprogression. This latter group had an inferior OS compared to patients without hyperprogression q (4.7 vs. 7.9 months).

## 7. Immune-Related Adverse Events in NSCLC

Patients with NSCLC being treated with ICI often experience the same common irAEs seen across all tumor types which often include dermatitis, colitis, and thyroid dysfunction. Though rarer, of particular interest in NSCLC is pneumonitis, as many patients have lower lung reserves due to pre-existing diseases like COPD or exposure to radiation therapy. In a retrospective study of 205 NSCLC patients on ICI, a higher rate of ICI pneumonitis of 19% was found, compared to the 3–5% previously reported in trials, and 53% of the pneumonitis case were grade 3–5 [78]. Prior chest radiation has been disproven to be a risk factor for ICI pneumonitis [79]. Time to ICI pneumonitis onset ranges from 1.5 to 127 weeks (median of 34 weeks) [80]. Unlike diffuse bilateral pneumonitis induced by targeted therapy, ICI pneumonitis may present more focally, sometimes with unilateral radiographic changes [81]. Patterns include chronic obstructive pneumonia-like, ground glass opacities, hypersensitivity type, and interstitial type [82].

Treatment of symptomatic pneumonitis is with corticosteroids (≥1 mg/kg), with a taper over 4–6 weeks. Pneumonitis is steroid responsive in >80% of cases [82]. However, chronic pneumonitis has been described, defined as pneumonitis for 12 weeks or more despite corticosteroids and ICI suspension, and warrants further immunosuppression [83]. In retrospective studies or case reports, success has been observed with the use of infliximab, intravenous immune globulin (IVIG), infliximab with IVIG, mycophenolate mofetil, and cyclophosphamide [80].

Overall, irAEs in NSCLC are associated with improved survival outcomes, particularly PFS [84,85]. However, a multivariate analysis of two retrospective studies has shown ICI pneumonitis in NSCLC to be associated with worse outcomes, though they did not examine whether the death was due to pneumonitis, disease progression, or other causes [86,87]. A retrospective study of NSCLC confirmed that pneumonitis, compared to other irAEs, was the most common toxicity associated with ICI suspension and steroid use (95% and 93% of cases, respectively) [88]. ICI rechallenge after pneumonitis resolution is challenging, given the risk of recurrence. Data on safety are limited. Two retrospective studies on ICI pneumonitis had small subgroups of patients rechallenged with ICI (*n* = 12, *n* = 16) and demonstrated a 32% recurrence rate [82,89].

Another emerging area of interest is the association of the microbiome with irAEs. A higher abundance of *Streptococcus*, *Paecalibacterium*, and *Stenotrophomonas* was associated with developing grade 3–5 irAEs [90]. In another study higher levels of *Dorea, Butyricicococcus,* and *Eubacterium ventriosum* were associated with grade 2–5 irAEs. In metagenomics profiling, alpha diversity was decreased in patients with grade 2–5 irAEs, with an overrepresentation of *Dorea, Anaerosporobacter mobilis, Butyricicococcus,* and *Enterococcus faecium* [91]. In future studies, it will be important to track microbiota changes during ICI therapy and at the time of irAEs. Additionally, it will be interesting to explore whether the respiratory microbiome may be correlated with pneumonitis.

## 8. Future Perspectives/Conclusions

ICI therapy has radically revolutionized the treatment of NSCLC. In the span of a decade, the field also matured quite rapidly. It is less likely that a novel ICI will bring a dramatic leap forward in the survival of cancer patients, as most targets have been extensively studied. Akin to what happened with the maturation of the field of chemotherapeutics, incremental gains will likely be achieved through optimization of the treatment schedule, finding the appropriate dose intensity, exploring biomarkers for efficacy and toxicity, and drawing solutions for the challenging patient populations (such as those with brain metastases or frail patients). A few fields, nevertheless, hold a lot of promise. The modulation of the microbiome or the blockade of inflammatory cytokines in conjunction with ICI still has the potential to yield impressive results. The results of those trials are eagerly awaited. Although it is not explored in this review, the basic science of immunotherapy is also a rapidly evolving field. Several key breakthroughs in the field of genomics, metagenomics, and special profiling have given us a glimpse into how the immune checkpoint blockade works in NSCLC. The clinical application of these findings will undoubtedly lead to further advancement in the field [92,93].

## Figures and Tables

**Table 1:** Summary of trials.

Study	Treatment (Treatment vs. Control Group)	Indication	Key Findings	Primary Endpoint
CheckMate 017 (NCT01642004) [4]	Nivo vs. Docetaxel.	Advanced SCC NSCLC that had PD during or after first-line chemo.	OS, RR, and PFS were significantly better with nivo than with docetaxel, regardless of PD-L1 expression level.	**OS** 9.2 vs. 6.0 months with doce. At 1 year, OS rate was 42% with nivo vs. 24% with doce. The RR was 20% with nivo vs. 9% with doce (*p* = 0.008). The expression of the PD-1 ligand (PD-L1) was neither prognostic nor predictive of benefit.
CheckMate 057 (NCT01673867) [4]	Nivo vs. Docetaxel.	Advanced Non-SCC NSCLC that had progressed during or after platinum-based doublet chemo.	OS was longer with nivo than with docetaxel.	**OS** 12.2 months in the nivo group and 9.4 months in the doce group (HR, 0.73; 96% CI, 0.59 to 0.89; *p* = 0.002). At 1 year, the OS rate was 51% with nivo vs. 39% with doce. With additional follow-up, the OS rate at 18 months was 39% with nivo vs. 23% with doce. The RR was 19% with nivo vs. 12% with doce (*p* = 0.02).
Keynote-010 (NCT01905657) [5]	Pembro vs. Docetaxel.	Previously treated, PD-L1-positive, advanced NSCLC.	Pembro prolongs OS and has a favorable benefit-to-risk profile in patients with previously treated, PD-L1-positive, advanced NSCLC.	**OS** significantly longer for pembro 2 mg/kg vs. doce (*p* = 0.0008) and for pembro 10 mg/kg vs. doce (*p* < 0.0001). Median PFS was 3.9 months with pembro 2 mg/kg, 4.0 months with pembro 10 mg/kg, and 4.0 months with doce, with no significant difference for pembro 2 mg/kg vs. doce (*p* = 0.07) or for pembro 10 mg/kg vs. doce (*p* = 0.004). In patients with at least 50% expressing PD-L1, OS was significantly longer with pembro 2 mg/kg than with doce (median 14.9 months vs. 8.2 months) and with pembro 10 mg/kg than with doce (17.3 months vs. 8.2 months; *p* < 0.0001).
OAK (NCT02008227) [6]	Atezo vs. Docetaxel.	Stage IIIB or IV SCC and non-SCC NSCLC that had received 1–2 previous treatment.	The first randomized phase 3 study that reported results of a PD-L1-targeted therapy, with atezo resulting in a clinically relevant improvement of OS vs. docetaxel in previously treated NSCLSC regardless of PD-L1 expression or histology.	OS significantly longer with atezo in the ITT and PD-L1-expression populations. In the ITT OS was improved with atezo compared with doce (median OS 13.8 months vs. 9.6 months; *p* = 0.0003). Patients in the PD-L1 low also had improved survival with atezo (median overall survival 12.6 months vs. 8.9 months. OS improvement was similar in patients with SCC in the atezo group or non-SCC.
Keynote-024 (NCT02142738) [7]	Pembro vs. Chemo (investigator’s choice of platinum-based chemo).	Previously untreated advanced NSCLC with at least 50% of PD-L1 expression and no EGFR or ALK mutation.	Pembro showed superior PFS compared to chemo.	PFS was 10.3 months (6.7 to not reached) in the pembro group vs. 6.0 months in the chemo group. The estimated rate of OS at 6 months was 80.2% in the pembro group vs. 72.4% in the chemo group.
Keynote-042 (NCT02220894) [7]	Pembro vs. Chemo (investigator’s choice of platinum-based chemo).	Previously untreated, PD-L1-expressing, locally advanced or metastatic NSCLC without EGFR or ALK mutation.	Pembro exhibited improved OS compared to chemo.	OS was significantly longer in the pembro group than in the chemo group. The median survival were 20 months for pembro vs. 12.2 months for chemo.
IMpower110 (NCT02409342) [8]	Atezo vs. Chemo.	Metastatic non-SCC or SCC NSCLC that had not previously received chemo and that had PD-L1 expression on at least 1%.	Atezo resulted in longer OS than platinum-based chemo in patients with NSCLC with high PD-L1 expression, regardless of histologic type.	OS was longer by 7.1 months in the atezo group than in the chemo group (20.2 months vs. 13.1 months; HR, 0.59; *p* = 0.01).
CheckMate 026 (NCT02041533) [9]	Nivo vs. Chemo.	Untreated stage IV or recurrent NSCLC and a PD-L1 expression of 1% or more.	Nivo was not associated with significantly longer PFS than chemo.	PD-L1 expression level of 5% or more, the median PFS was 4.2 months with nivo vs. 5.9 months with chemo (*p* = 0.25), and the median OS was 14.4 months vs. 13.2 months.
MYSTIC (NCT02453282) [10]	Durva +/− Tremelimumab vs. Standard Chemo.	First-line treatment of Metastatic NSCLC.	The study did not meet primary end points of improved OS with durva vs. chemo or improved OS or PFS with durva plus tremelimumab vs. chemo in patients with ≥25% of tumor cells expressing PD-L1.	Exploratory analyses identified a bTMB threshold of ≥20 mutations per megabase for optimal OS benefit with durvalumab plus tremelimumab.
JAVELIN Lung 200 (NCT02395172) [11]	Avelumab vs. docetaxel.	Patients with platinum-treated advanced NSCLC.	No significant difference observed.	In patients with PD-L1-positive tumors, median OS did not differ significantly between avelumab and docetaxel groups (11.4 months [95% CI 9.4–13.9] vs. 10.3 months [8.5–13.0]).
CheckMate 227 (NCT02477826) [12]	Nivo + Ipi vs. Chemo.	Stage IV or recurrent NSCLC and a PD-L1 expression level of 1%.	First-line treatment with nivo plus ipi resulted in a longer duration of OS than did chemo in patients with NSCLC, independent of the PD-L1 expression level.	Patients PD-L1 expression of 1% or more, the median duration of OS was 17.1 months with nivo plus ipi and 14.9 months with chemo (*p* = 0.007), with 2-year OS rates of 40.0% and 32.8%, respectively.
NEPTUNE (NCT02542293) [13]	Durva plus Tremelimumab.	First-line metastatic NSCLC with TMB ≥ 20 mutations/Mb.	The study did not meet the primary endpoint for OS.	OS with durva plus tremelimumab vs. chemo did not reach statistical significance, median OS (11.7 vs. 9.1 months); the HR for PFS was 0.77 (95% confidence interval, 0.51–1.15; median PFS, 4.2 vs. 5.1 months).
Keynote189 (NCT02578680) [14]	Pembro + Chemo.	Metastatic non-SCC NSCLC, without EFGR or ALK mutations.	The addition of pembro to chemo of pemetrexed and a platinum-based drug resulted in significantly longer OS and PFS than chemo alone.	OS was 69.2% in the pembro-combination group vs. 49.4% in the placebo-combination group (HR, 0.49; 95% CI, 0.38 to 0.64; *p* < 0.001). Improvement in OS was seen across all PD-L1 categories. Median PFS was 8.8 months in the pembro combination group and 4.9 months in the placebo-combination group.
IMpower130 (NCT02367781) [15]	Atezo in combination with carboplatin plus nab-paclitaxel chemo compared with chemo alone.	First-line treatment for metastatic non-SCC NSCLC.	Improvement in OS and a significant improvement in PFS with atezo plus chemo vs. chemo as first-line in patients with stage IV and no ALK or EGFR mutations.	Significant improvements in OS (18.6 months [95% CI 16.0–21.2] in the atezo plus chemo group and 13.9 months [12.0–18.7] in the chemo group; [HR] 0.79 [95% CI 0.64–0.98]; *p* = 0.033) and median PFS (7.0 months [95% CI 6.2–7.3] in the atezo plus chemo group and 5.5 months in the chemo group).
IMpower131 (NCT02367794) [16]	Atezo in combination with Carboplatin and Nab-Paclitaxel.	Advanced SCC NSCLC.	Adding atezo to platinum-based chemo significantly improved PFS in patients with first-line SCC NSCLC; OS was similar between the arms.	PFS improvement with A + CnP versus CnP was seen in the ITT population (median, 6.3 versus 5.6 mo; hazard ratio [HR] = 0.71, 95% confidence interval [CI]: 0.60–0.85; *p* = 0.0001).
IMpower132 (NCT02657434) [17]	Atezo + Chemo carboplatin or cisplatin plus pemetrexed (PP) or APP.	Chemo-naive patients with stage IV non-SCC NSCLC without EGFR or ALK mutations.	The study met co-primary PFM end point but not OS end point.	Significant PFS improvement vs. PP (*p* < 0.0001). OS for the APP group was numerically better but not statistically significant at the interim (22 May 2018; median = 18.1 versus 13.6 mo.) and final analyses (18 July 2019; median = 17.5 vs. 13.6 mo.)
IMpower150 (NCT02366143) [18]	Atezo plus carboplatin plus paclitaxel (ACP), beva plus carbo plus paclitaxel (BCP), or atezo plus BCP (ABCP).	Metastatic non-SCC NSCLC with EGFR/ALK mutations.	The addition of atezo to beva plus chemo significantly improved PFS and OS regardless of PD-L1 expression and EGFR or ALK status.	PFS was longer in the ABCP group than in the BCP group (8.3 months vs. 6.8 months); PFS was also longer in the ABCP group than in the BCP group in the entire intention-to-treat population.
ATTLAS (NCT03991403) [19]	Atezo plus Beva and Chemo (pemetrexed plus carboplatin or cisplatin).	EGFR or ALK-mutated NSCLC after TKI therapy. Metastatic (second line).	Atezo in combination with beva and chemo demonstrated improved RR and PFS, but similar OS.	PFS benefit, ORR were higher for patients treated with ABCP compared with chemo (69.5% versus 41.9%, respectively; *p* < 0.001).
Keynote 407 (NCT02775435) [20]	Pembro + platinum-based chemo.	Standard first-line therapy for metastatic, SCC NSCLC.	The addition of pembro to chemo resulted in significantly longer OS and PFS than chemotherapy alone.	Median OS was 15.9 months (95% confidence interval [CI], 13.2 to not reached) in the pembro-combination group and 11.3 months in the placebo-combination group (hazard ratio for death, 0.64; 95% CI, 0.49 to 0.85; *p* < 0.001).
CheckMate 9LA (NCT03215706) [21]	Nivo plus ipi combined with two cycles of chemo.	First-line metastatic NSCLC.	Significant improvement in OS vs. chemo alone and had a favorable risk–benefit profile.	OS was significantly longer in the experimental group than in the control group (median 14.1 months [95% CI 13.2–16.2] vs. 10.7 months [9.5–12.4]; hazard ratio [HR] 0.69 [96.71% CI 0.55–0.87]; *p* = 0.00065).
POSEIDON (NCT03164616) [22]	Durva +/− Tremelimumab in combination with chemo.	First-line metastatic NSCLC.	D + CT significantly improved PFS versus CT. A limited course of tremelimumab added to durva and chemo significantly improved OS and PFS vs. CT.	PFS improved with D + CT vs. CT (hazard ratio [HR], 0.74; 95% CI, 0.62 to 0.89; *p* = 0.0009; median, 5.5 vs. 4.8 months); a trend for improved OS did not reach statistical significance PFS, 6.2 vs. 4.8 months and OS 14.0 vs. 11.7 months.
CheckMate 153 (NCT02066636) [23]	Continuous vs. 1 y fixed-duration Nivolumab.	Previously treated advanced NSCLC.	Continuous treatment with nivo beyond 1 year conferred a significant survival advantage.	Follow-up of 13.5 months, median PFS was longer with continuous vs. 1-year fixed-duration treatment (PFS population: 24.7 months vs. 9.4 months; [HR], 0.56 [95% CI, 0.37 to 0.84]).
Keynote001 (NCT01295827) [24]	Pembro.	Advanced NSCLC.	Pembro had an acceptable side-effect profile and showed antitumor activity. PD-L1 expression in at least 50% correlated with improved efficacy of pembro.	ORR was 19.4%, and the median duration of response was 12.5 months. The median duration of PFS was 3.7 months, and the median duration of OS was 12.0 months.
CheckMate 816 (NCT02998528) [25]	Stage IB to IIIA resectable NSCLC to receive nivo plus platinum-based chemo or platinum-based chemo alone, followed by resection.	Neoadjuvant.	Neoadjuvant nivo plus chemo resulted in significantly longer EFS and a higher % of patients with a pCR than chemo alone. The addition of nivo to neoadjuvant chemo did not increase the incidence of AEs or impede the feasibility of surgery.	The median EFS was 31.6 months (95% confidence interval [CI], 30.2 to not reached) with nivo plus chemo and 20.8 months (95% CI, 14.0 to 26.7) with chemo alone (HR for disease progression, disease recurrence, or death, 0.63; 97.38% CI, 0.43 to 0.91; *p* = 0.005). The % of patients with a pCR was 24.0% (95% CI, 18.0 to 31.0) and 2.2%. (95% CI, 0.6 to 5.6), respectively (odds ratio, 13.94; 99% CI, 3.49 to 55.75; *p* < 0.001).
Keynote091/PEARLS (NCT02504372) [26]	Pembro vs. placebo as adjuvant therapy for completely resected stage IB–IIIA NSCLC.	Adjuvant therapy.	Pembro significantly improved DFS compared with placebo and was not associated with new safety signals in completely resected, PD-L1-unselected, stage IB–IIIA NSCLC.	Median DFS was 53.6 months (95% CI 39.2 to not reached) in the pembro group vs. 42.0 months (31.3 to not reached) in the placebo group (HR 0.76 [95% CI 0.63–0.91], *p* = 0.0014). In the PD-L1 TPS of 50% or greater population, median DFS was not reached in either the pembro group (95% CI 44.3 to not reached) or the placebo group (95% CI 35.8 to not reached; HR 0.82 [95% CI.0·57–1.18]; *p* = 0.14).
CheckMate 77T (NCT04025879) [27]	Neoadjuvant nivo plus chemo followed by adjuvant nivo in untreated resectable stage IIA–IIIB.	Perioperative regimen followed by surgery and adjuvant nivo.	Statistically significant and clinically meaningful improvement in EFS compared with neoadjuvant chemo plus placebo followed by surgery and adjuvant placebo in patients with resectable stage IIA to IIIB NSCLC.	Significantly improved median EFS compared with chemo plus adjuvant placebo (not reached vs. 18.4 months; HR 0.58; 97.36% confidence interval [CI] 0.42–0.81; *p* = 0.00025).
AEGEAN (NCT03800134) [28]	Neoadjuvant platinum-based chemo plus durva vs. placebo, followed by adjuvant durva or placebo.	Resectable stage II to IIIB NSCLC.	Statistically significant improvement in EFS with addition of durva to neoadjuvant chemo, followed by adjuvant durva.	Significantly improved median EFS compared with chemo plus adjuvant placebo (NR vs. 25.9 months; HR 0.68; 95% confidence interval [CI] 0.53–0.88; *p* = 0.004).
KEYNOTE-671 (NCT03425643) [29]	Neoadjuvant platinum-based chemo plus pembro or placebo, followed by adjuvant pembro or placebo.	Resectable stage II to IIIB NSCLC.	Statistically significant improvements in EFS and OS with neoadjuvant chemo plus pembro then adjuvant pembro	Significant improvements in median EFS (NR vs. 17 months; HR 0.58; 95% CI, 0.46–0.72) and median OS (NR vs. 52.4 months; HR 0.72; 95% CI, 0.56–0.93, *p* = 0.00517).
Neotorch (NCT04158440) [30]	Neoadjuvant platinum-based chemo plus toripalimab or placebo, followed by adjuvant toripalimab or placebo.	Resectable stage II or III NSCLC without EGFR or ALK alterations.	Statistically significant improvement in EFS with neoadjuvant chemo plus toripalimab then adjuvant toripalamab.	Significant improvements in median EFS (NR vs. 15.1 months; HR 0.4, 95% CI, 10.6–21.9; *p* < 0.001).

## Data Availability

Not applicable.
